# Percutaneous reduction with double screwdrivers versus limited open reduction in the treatment of irreducible extracapsular hip fractures

**DOI:** 10.1186/s12891-022-05390-x

**Published:** 2022-05-06

**Authors:** Qiang Huang, YiBo Xu, HanZhong Xue, Qian Wang, Ming Li, Cheng Ren, Yao Lu, Zhong Li, Kun Zhang, Teng Ma

**Affiliations:** Department of Orthopedic Surgery, Xi’an Jiaotong University, Hong Hui hospital, Xi’an, 710054 Shaanxi China

**Keywords:** Extracapsular hip fracture, Irreducible, Screwdriver, Percutaneous reduction

## Abstract

**Background:**

The reduction in irreducible extracapsular hip fractures has always been controversial. Here, we present a new minimally invasive reduction technique and compare it with limited open reduction (LOR) to treat irreducible extracapsular hip fractures.

**Methods:**

From January 2016 to January 2018, our institution treated 653 patients with extracapsular hip fractures by intramedullary fixation. Among them, 525 cases got a successful closed reduction. The other 128 were irreducible and reduced by percutaneous reduction with double screwdrivers (PRDS) or LOR. There were 66 cases in the PRDS group while 62 in the LOR group. All fractures were classified using the Evans-Jensen classification. In addition, the differences in incision length, blood loss, fluoroscopic number, operation time, inpatient time, weight training time, Harris score, and complications were analyzed.

**Results:**

The incision length was 8.4 ± 1.4 cm in the PRDS group and 15.3 ± 3.0 cm in the LOR group, respectively (*p* < 0.05); blood loss was equal to 151 ± 26 and 319 ± 33 ml, respectively (*p* < 0.05); fluoroscopic number was 14 ± 3 and 8 ± 2, respectively (*p* < 0.05); operation time was 44 ± 9 and 73 ± 11 min, respectively (*p* < 0.05); inpatient time was 6.2 ± 1.7 and 8.4 ± 2.2 days, respectively (*p* < 0.05); weight training time after the operation was 4.5 ± 1.5 and 10.7 ± 1.8 days, respectively (*p* < 0.05); and the excellent rate of Harris score was 92.4% and 88.7%, respectively (*p* > 0.05). There was no significant difference in complication incidence between the two groups (*p* > 0.05).

**Conclusions:**

The PRDS group presented better clinical effects for managing irreducible extracapsular hip fractures than the LOR. Therefore, the PRDS technique could be the first reduction choice for patients with irreducible fractures.

## Background

Extracapsular hip fractures involve the area between the greater and lesser trochanter [1]. The incidence of extracapsular hip fractures in the elderly increases every year, and the mortality within one year after this fracture could be as high as 15 to 20% [2, 3]. This puts both surgeons and patients under tremendous pressure. Intramedullary fixation is one of the primary methods for managing such fractures. Clinical practice indicates that longitudinal traction and appropriate rotation of the fractured extremity will result in an acceptable closed reduction of most extracapsular hip fractures [4].

**Fig. 1:**
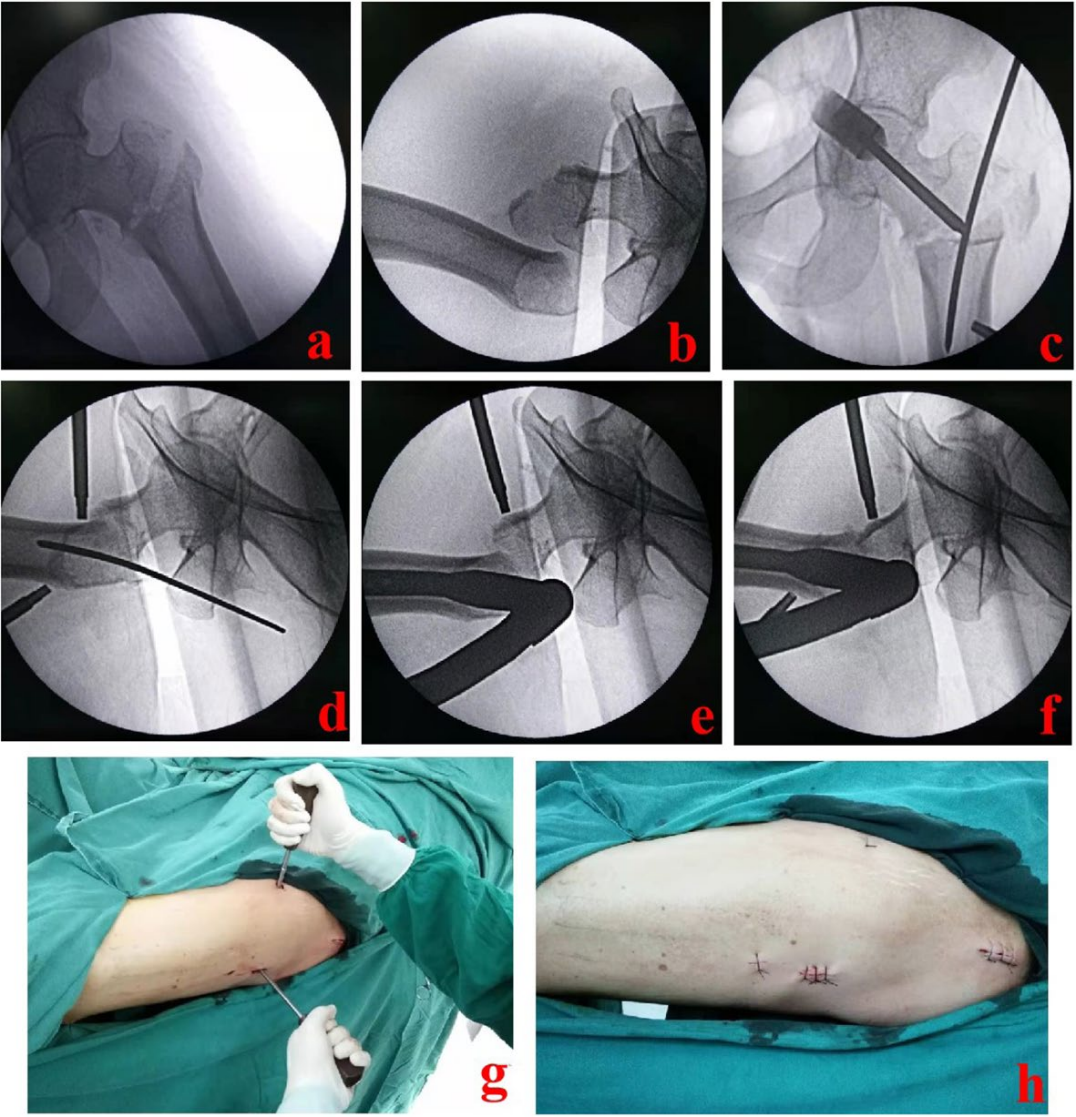
A 63-year-old female suffered from an extracapsular hip fracture and was reduced by the PRDS technique. **a** and **b** After repeated closed reduction, the fracture fragments were still dislocated obviously; **c-f**: Under fluoroscopic guidance, the minimally invasive PRDS technique was performed; **g** Schematic diagram of the PRDS technique; **h** Sketch of incisions. PRDS stands for percutaneous reduction with double screwdrivers

"Irreducible fractures" are extracapsular hip fractures that cannot be reduced successfully. Reducing these irreducible extracapsular hip fractures has always been a controversial issue. Several reports recommended open reduction when encountering irreducible fractures [5, 6]. However, open reduction brings significant trauma and a large amount of blood loss. This may lead to many complications, such as infection, nonunion, and others [7, 8]. Currently, most elderly patients with extracapsular hip fractures are often accompanied by cardiovascular and cerebrovascular diseases. As a result of these circumstances, some patients are unable to sustain severe surgical trauma, which may result in an increase in perioperative mortality. Others have explored different minimally invasive reduction techniques. During surgery, the auxiliary tools used include Steinman pins, bone hooks, pointed reduction clamps, and others [9–11]. Based on our clinical experience, we developed a relatively simple reduction method by using double screwdrivers as joysticks (Fig. 1). The screwdrivers are percutaneously inserted to control the distal and proximal fragments. According to the fragment's dislocation status, they could play different roles, such as jacking, pressing, lifting, prying, or pushing. The PRDS technique has not been reported yet to the best of our knowledge. Our study is the first report on this new technique.

Here, we aimed to present our experience of PRDS and compare it with the LOR technique for the treatment of irreducible extracapsular hip fractures. Therefore, it was analyzed retrospectively and reported as follows.

## Methods

### Inclusion criteria

(1) Only an extracapsular hip fracture occurred; (2) Irreducible fractures; (3) Patients with complete medical records.

### Exclusion criteria

(1) Polytrauma patients; (2) Reducible fractures; (3) Open fractures; (4) Patients who died within one year after operation; (5) Patients with severe medical diseases unable to tolerate anesthesia or surgery; (6) Patients with incomplete medical records.

### General information

From January 2016 to January 2018, 128 patients with irreducible extracapsular hip fractures at Xi'an Hong Hui hospital were included in the study. All patients were treated by intramedullary fixation. There were 58 males and 70 females. The participants varied in age from 22 to 95 years old, with the majority being senior patients. All fractures were classified using the Evans-Jensen classification [12]. Sixty-six cases were reduced by the PRDS technique while 62 by LOR. In addition, the preoperative physiological status of these patients was evaluated using the American Society of Anesthesiologists grading standard (ASA) score [13].

### Preoperative treatment

All patients were given general examinations after admission. Anteroposterior (AP) and lateral X-ray pictures of the damaged hip were included in the special tests. Atomization inhalation was routinely used to protect the lungs. Venous color Doppler ultrasound was used to check veins of both lower limbs. Before the procedure, patients or their family members completed an informed consent form.

### Surgical procedures

#### PRDS group

**Fig. 2:**
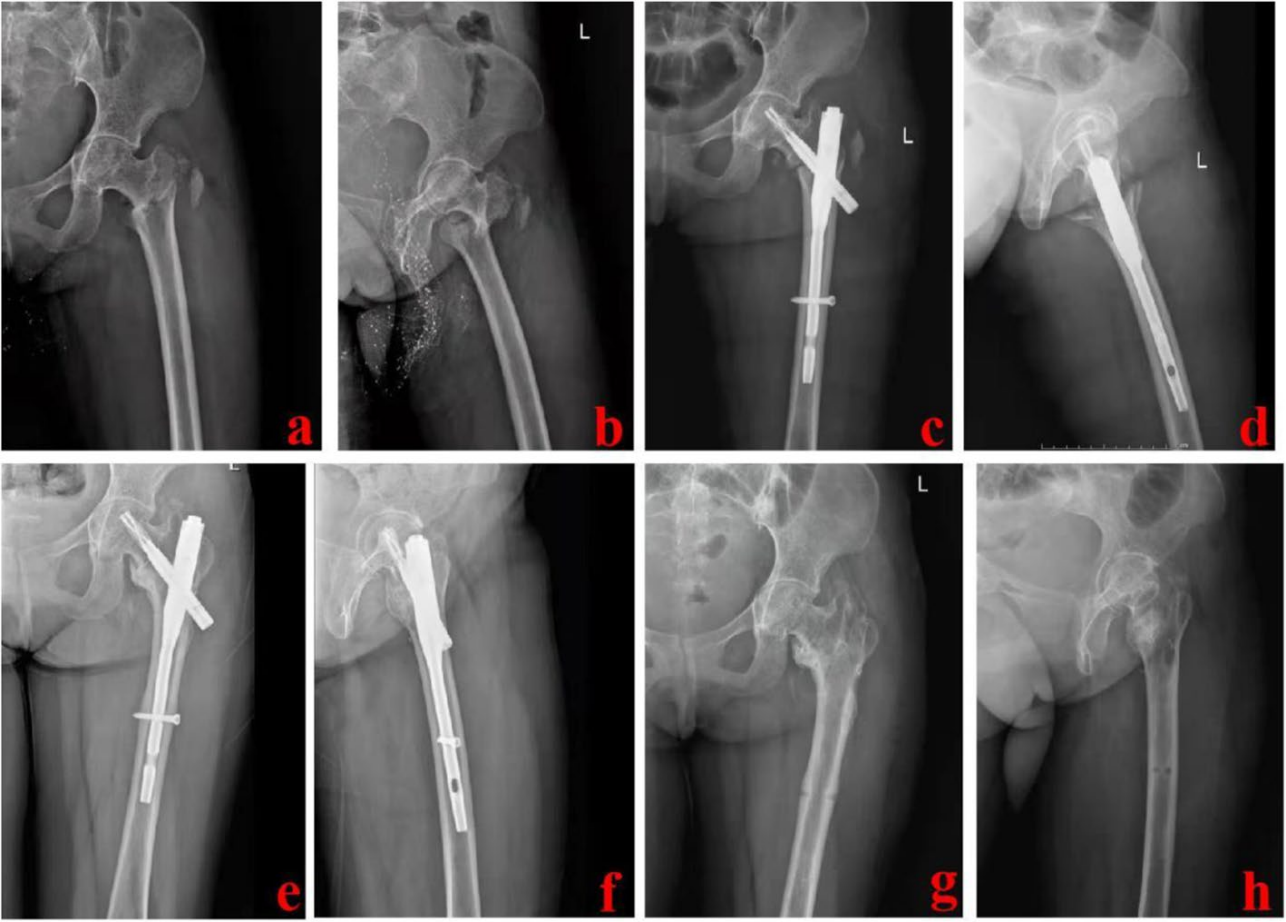
Follow-up image data of the 63-year-old female. **a** and **b** AP and lateral images of the hip joint before operation; **c** and **d** Postoperative X-ray images showed good reduction and fixation; **e** and **f** One year after the operation, the fracture healed well; **g** and **h** One and a half years after the operation, the internal fixation device was taken out. AP stands for anteroposterior

**Fig. 3:**
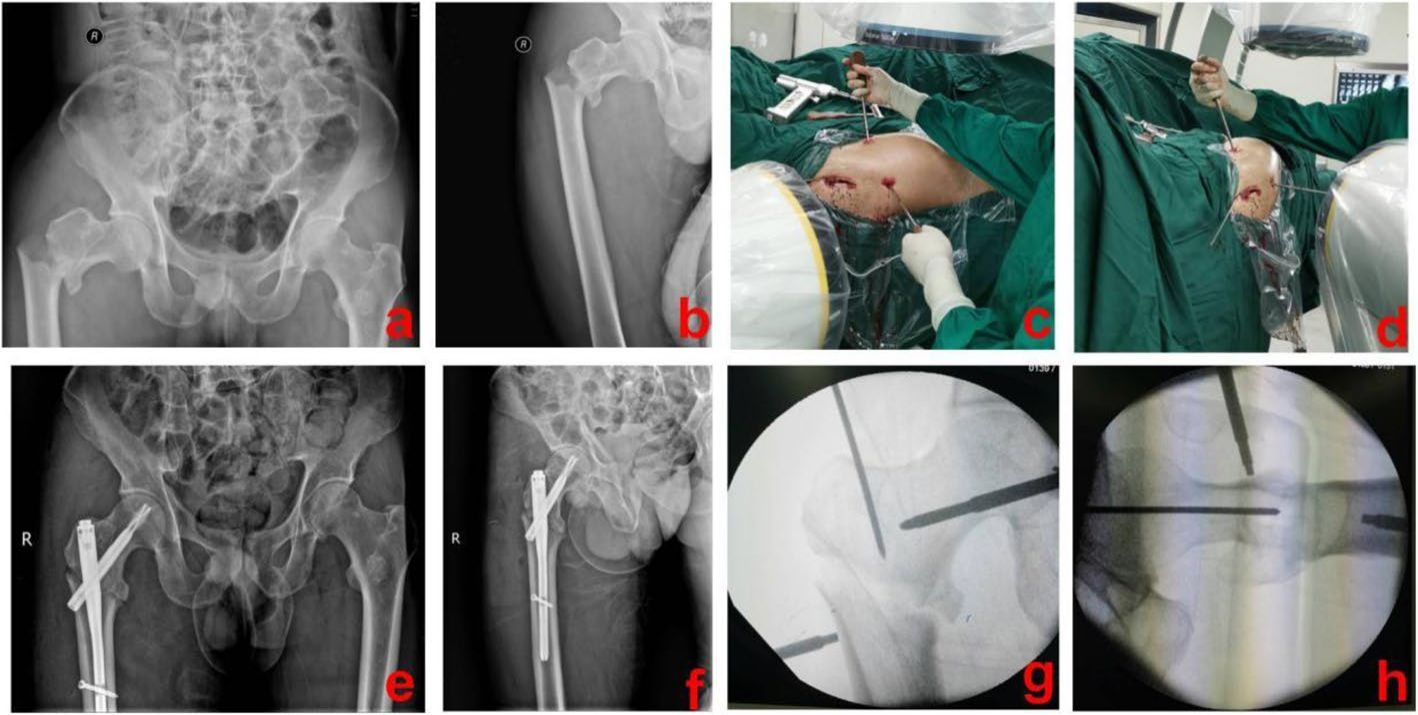
A 47-year-old male suffered from an extracapsular hip fracture and was treated using the PRDS technique. **a** and **b** Preoperative X-ray images of the injured hip; **c** and **d** During operation, PRDS technique was used for reduction; **e** and **f** Postoperative X-ray images showed that this fracture was well reduced and fixed; **g** and **h** Intra-operative X-ray images of the PRDS technique. PRDS stands for percutaneous reduction with double screwdrivers

**Fig. 4:**
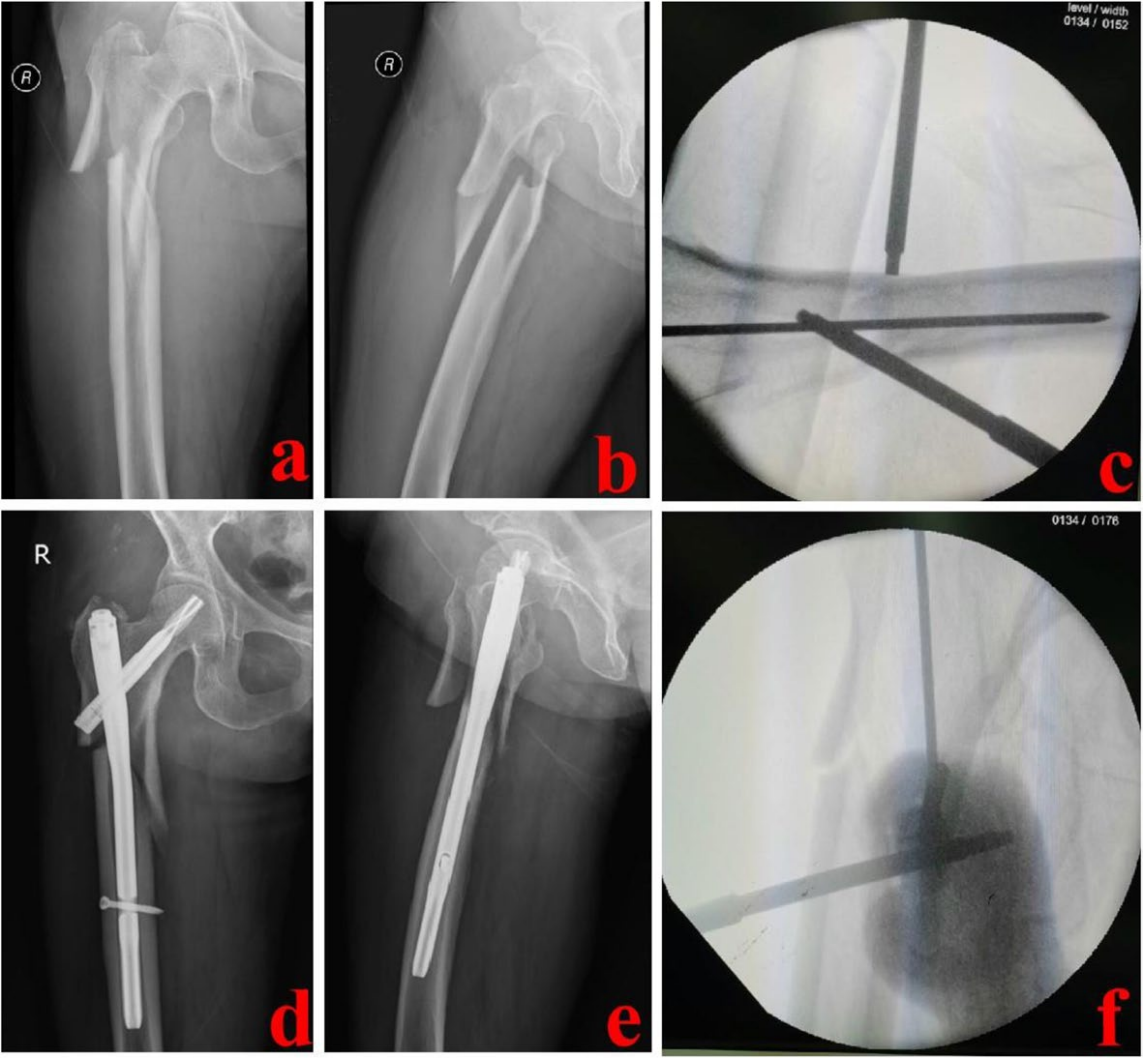
A 61-year-old patient was successfully treated with the PRDS technique. **a** and **b**: Preoperative X-ray images of the injured hip; **c** and **f** Intra-operative images of the PRDS technique; **d** and **e** Postoperative X-ray images of the affected hip. PRDS stands for percutaneous reduction with double screwdrivers

The patient was placed in a supine position and fixed on the traction table. Under G-arm fluoroscopic guidance [14], the limb length was restored by longitudinal traction. Next, the rotational dislocation was corrected by rotating the affected limb. When repeated closed reduction failed, the chief surgeon decided to use the PRDS technique. The patient was prepared and draped. The entry point of the main nail was confirmed and opened. Then, the fracture site was confirmed and marked under fluoroscopic guidance. One screwdriver was introduced percutaneously through a 5-mm incision made in front of the proximal plane of the fracture. In the AP and lateral images, the screwdriver tip was placed near the anterior cortex of the proximal piece. The second screwdriver was introduced percutaneously on the outside of the distal fragment. The screwdriver tip was positioned 1–2 cm proximal to the center of the fracture line at the distal fragment. According to the fragment displacement, the two screwdrivers were used as joysticks to assist in reduction. The proximal fragment was usually tilted forward and could be pressed down with the front screwdriver. The distal fragment was displaced posteriorly and could be lifted upward by the lateral screwdriver. If the distal fragment was displaced to the lateral side, the lateral screwdriver should be placed on the lateral cortex and pushed inward. The chief surgeon should keep the reduction using screwdrivers for a little time after it was adequate. Simultaneously, the assistant inserted the main nail into the medullary cavity. The cephalomedullary nail and distal locking screws were then inserted one after the other. Typical cases are shown in Figs. 2, 3 and 4.

#### LOR group

**Fig. 5:**
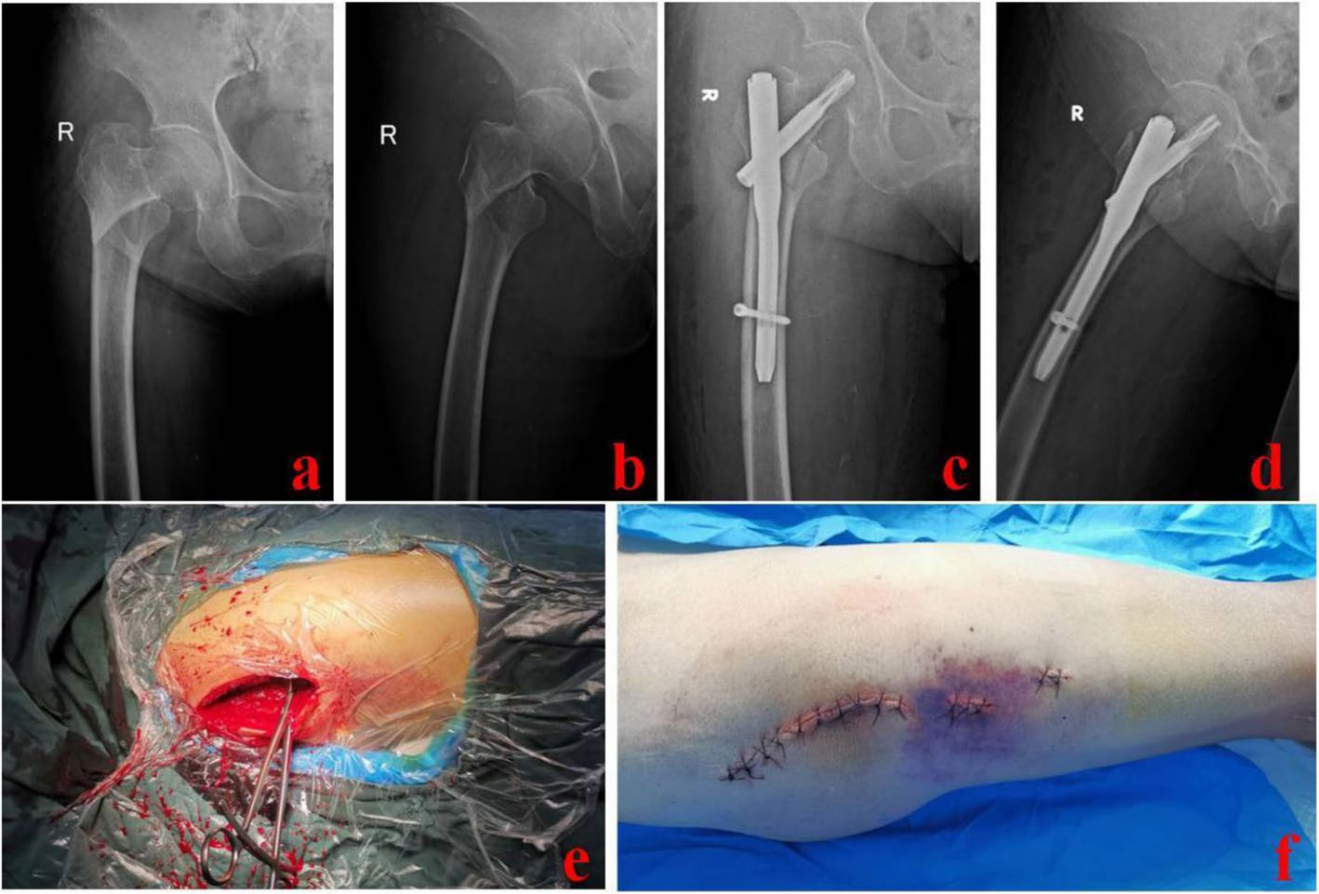
A 73-year-old female was treated with the LOR technique. **a** and **b** Preoperative X-ray images of the injured hip; **c** and **d** Postoperative X-ray images; **e**: Clamp reduction was performed during operation; **f** Schematic diagram of the incisions. LOR stands for limited open reduction

The LOR technique was used after closed reduction failed. A lateral longitudinal incision of 11–12 cm was made with the greater trochanter as the center. The apex of the greater trochanter and fragments were exposed appropriately. Hematoma and clots were removed. Displaced fragments were reduced by manipulative reduction, clamping, and others. Then, an appropriate intramedullary nail was selected and inserted under fluoroscopic guidance. A typical case is shown in Fig. 5.

### Postoperative treatment

Patients were given atomization inhalation and anticoagulant therapy regularly. Attention should be paid to anti-osteoporosis treatment. Patients were advised to sit up and flip over on their own as soon as possible after being coached through active and passive functional exercises. Early after the operation, weight-bearing training was gradually carried out under the protection of a walking aid.

### Observation indexes

We looked at differences in incision length, blood loss, fluoroscopic number, operation time, inpatient time, weight training time, and Harris score [15]. Weight training time refers to the time from the completion of the operation to the beginning of walking with a walking aid. Complications were compared between the two groups, including nonunion, incision infection, sacral ulcer, pneumonia, and deep venous thrombosis (DVT). All patients were followed for at least one year.

### Statistical analysis

SPSS 23.0 software (IBM Company, USA) was used to process data, and measurement data were expressed as the mean ± standard deviation. The unpaired *t*-test was used for comparisons between the two groups, including age, BMI, incision length, blood loss, fluoroscopic number, operation time, inpatient time, and weight training time. Count data were analyzed using the χ2 test, comprising gender, ASA score, classification, excellent rate of Harris score, and postoperative complications. *p* < 0.05 was considered statistically significant.

## Results

### Demographics of the two groups

As shown in Table 1, 30 males and 36 females were in the PRDS group, while 28 males and 34 females were in the LOR group. The mean age was 68 ± 16 and 66 ± 12 years in the PRDS and the LOR groups, respectively; and the mean body mass index (BMI) was 23 ± 4 and 24 ± 3 kg/m^2^, respectively. Based on the ASA score, there were 7 patients classified as grade I, 30 as grade II, 25 as grade III, and 4 as grade IV cases in the PRDS group, while 6 as grade I, 24 as grade II, 27 as grade III, and 5 as grade IV patients in the LOR group. According to the Evans-Jensen classification, the PRDS group included 5 patients classified as type II, 20 type III, 22 type IV, and 19 type V fractures, while the LOR group comprised 3 as type II, 16 type III, 23 type IV, and 20 type V fractures. There was no statistical difference in demographic information between the two groups (*p* > 0.05, Table 1).

**Table 1:** Demographics of patients with extracapsular hip fractures

Variable	PRDS group (*n* = 66)	LOR group (*n* = 62)	*p* value
Gender			0.973
male	30	28	
female	36	34	
Age (year)	68 ± 16	66 ± 12	0.427
BMI (kg/m^2^)	23 ± 4	24 ± 3	0.114
ASA score			0.848
grade I	7	6	
grade II	30	24	
grade III	25	27	
grade IV	4	5	
Evans-Jensen classification			0.833
type II	5	3	
type III	20	16	
type IV	22	23	
type V	19	20	

*PRDS* stands for percutaneous reduction with double screwdrivers, *LOR* stands for limited open reduction, *BMI* stands for body mass index, *ASA* stands for American society of anesthesiologists

### Comparison of operation indexes and clinical effects

As shown in Table 2, the mean incision length was 8.4 ± 1.4 cm in the PRDS group and 15.3 ± 3.0 cm in the LOR group (*p* < 0.05). The mean blood loss was 151 ± 26 ml in the PRDS group and 319 ± 33 ml in the LOR group (*p* < 0.05). Additionally, the mean fluoroscopic number was 14 ± 3 and 8 ± 2, with a significant difference between the two groups (*p* < 0.05). The mean operation time was 44 ± 9 and 73 ± 11 min, also with a significant difference between the two groups (*p* < 0.05). The mean inpatient time was 6.2 ± 1.7 and 8.4 ± 2.2 days, respectively (*p* < 0.05); and the mean weight training time after the operation was 4.5 ± 1.5 and 10.7 ± 1.8 days (*p* < 0.05). The excellent rate of Harris scores was 92.4% and 88.7%, with no statistically significant difference between the two groups (*p* > 0.05).

**Table 2:** Operation indexes and clinical effects of the PRDS and LOR technique

Variable	PRDS group (*n* = 66)	LOR group (*n* = 62)	*p* value
Incision length (cm)	8.4 ± 1.4	15.3 ± 3.0	*p* < 0.05
Blood loss (ml)	151 ± 26	319 ± 33	*p* < 0.05
Fluoroscopic number (N.)	14 ± 3	8 ± 2	*p* < 0.05
Operation time (min)	44 ± 9	73 ± 11	*p* < 0.05
Inpatient time (d)	6.2 ± 1.7	8.4 ± 2.2	*p* < 0.05
Weight training time (d)	4.5 ± 1.5	10.7 ± 1.8	*p* < 0.05
Excellent rate of Harris score	61/66 (92.4%)	55/62 (88.7%)	0.471

### Comparison of complications between the two groups

As shown in Table 3, postoperative complications included sacral ulcer in one patient, pneumonia in three, and DVT in three for the PRDS group. In the LOR group, complications included nonunion in two patients, incision infection in two individuals, sacral ulcer in five patients, pneumonia in six patients, and DVT in five patients. There was no significant difference in complication incidence between the two groups (*p* > 0.05, Table 3). All postoperative complications in the two groups were actively treated and cured.

**Table 3:** Postoperative complications of the PRDS and LOR technique

Variable	PRDS group (*n* = 66)	LOR group (*n* = 62)	*p* value
Nonunion n(%)	0 (0.0%)	2 (3.2%)	-
Incision infection n(%)	0(0.0%)	2 (3.2%)	-
Sacral ulcer n(%)	1(1.5%)	5(8.1%)	0.182
Pneumonia n(%)	3(4.5%)	6(9.7%)	0.430
DVT n(%)	3(4.5%)	5(8.1%)	0.648

*DVT* stands for deep vein thrombosis

## Discussion and conclusions

The number of older people suffering from extracapsular hip fractures is rising as the population ages [16, 17]. This poses a great challenge to trauma surgeons. Reducing surgical invasiveness, achieving biomechanical stability, and encouraging early weight-bearing are all important considerations in the effective treatment of this fracture in the elderly.

In most extracapsular hip fractures, the proximal fragment is prized forward, and the distal fragment is displaced posteriorly [18]. All or part of the lesser trochanter is connected to the proximal fragment. Moreover, the proximal fragment is in flexion, abduction, and external rotation due to iliopsoas and external rotator muscle tension. Meanwhile, the distal fragment is located on the posteromedial side. The lateral view shows that the separation between proximal and distal fragments is large [9, 10]. Sometimes the iliopsoas tendon is even embedded into the broken site. Some of these fractures can not be reduced closely. Said et al. [5] reported a rare type of irreducible fracture. The distal fragment included the lesser trochanter in their study, and there was a long spike on the head-neck fragment. The AP view revealed upward riding of the distal fragment, while the lateral view showing the distal fragment in front of the head and neck. The procedures for reducing these irreducible fractures have long been a contentious topic.

Open reduction was widely used formerly. For patients with irreducible fractures, a satisfactory reduction could be achieved by open reduction. The increased trauma and problems induced by open reduction, on the other hand, cannot be overlooked. With the promotion of the concept of accelerated rehabilitation, trauma surgeons are keen to explore minimally invasive techniques. Several reports have applied minimally invasive reduction techniques to manage irreducible extracapsular hip fractures. Chun et al. [9] used one or two Steinman pins as a joystick in sagittally unstable extracapsular hip fractures. The 4.2-mm Steinmann pin was introduced through a 2-mm-stab wound made at the fracture site. The operator could push or lever the cortex with the pin to reduce displaced fragments. Nevertheless, there was some concern regarding the potential problems with the above technique, such as lateral femoral cutaneous nerve injury, inadvertent vascular puncture, and adding comminution. Moreover, Kim et al. [10] suggested that the bone hook technique could be used in some specific patterns of extracapsular hip fractures. They did not have to make a new incision to implant the bone hook since they could utilize the same one they used to place the nail. The bone hook was made to slide along the anterior cortex of the proximal femur lying in close contact with the bone and then dipped into the fracture site. However, inserting a bone hook into the fracture site required some skill. When the bone hook is inserted, the important anterior femoral neurovascular structures should not be injured. Yoon et al. [11] used a pointed reduction clamp as a tool for the percutaneous reduction of these fractures. Their technique proved to be useful since it was less invasive, maintained reduction throughout the procedure, and significantly reduced the surgical time compared with the classical open reduction. However, a little incision was required when inserting the pointed reduction clamp. The above reduction tools have sharp tips, which may aggravate soft tissue injury and even damage neurovascular structures. Moreover, in the above studies, only specific irreducible fractures were selected, and the number of patients included was small. It was unclear whether these reduction techniques could work well for another type of irreducible extracapsular hip fractures.

The PRDS approach was created by the authors specifically for patients with irreducible extracapsular hip fractures. The first screwdriver is inserted in the proximal fragment's front, while the second is inserted on the distal fragment's exterior. The double screwdrivers usually work in mutually perpendicular directions. The proximal fragment is pressed posteriorly by the front screwdriver during surgery, and the distal fragment is pushed inward or anteriorly with the lateral screwdriver. Some of these fractures are displaced in different directions, such as the type in Said's study [5]. For these special cases, the insertion of double screwdrivers can be designed in advance to assist in reduction successfully. In our study, 66 cases were treated by the PRDS technique. These fractures showed different dislocations, and all could be reduced minimally with the assistance of double screwdrivers. Based on our results and experience, the PRDS technique displayed some advantages. Compared with the LOR group, the PRDS group showed smaller incisions, less blood loss, shorter operation time, and less inpatient time. The PRDS technique is a minimally invasive percutaneous reduction technique in essence. This technique can enable patients to begin functional exercises and bear weight early. As a result, rehabilitation will be accelerated, and patients will be able to return to regular life and work sooner. Moreover, the screwdriver tip is blunt, which will not cause additional soft tissue damage, especially accidental puncture of neurovascular structures. It is introduced percutaneously, so it has little interference with the local circulation of the fracture site. As we know, good circulation is an important factor for incision and fracture healing. Our new minimally invasive reduction technique is beneficial in protecting the local circulation. Moreover, the screwdriver is easy to obtain, and the method is relatively simple, which is very suitable for broader promotion.

### Limitations

There are still some limitations in this research. We compared a minimally invasive reduction technique with limited open reduction, not the comparison of different minimally invasive reduction techniques. This may lead to some deviation. Although the same group of surgeons performed all operations, we used limited open reduction in the early stage of this period to manage irreducible fractures. With the accumulation of experience, we explored this minimally invasive reduction technique (PRDS technique). Therefore, we applied the PRDS technique more frequently in the later stage of this period. This would also result in some deviation. Yet, as this was a single-center retrospective study, these deviations would not interfere with our conclusions. We will conduct a multi-center randomized controlled study in further research. The number of cases included was small, and the follow-up time was short. We will actively improve in the future.

## Conclusions

PRDS and LOR are two effective reduction methods for treating patients with irreducible extracapsular hip fractures. The limb function score and complication incidence were generally similar between the two groups. However, the PRDS technique is less invasive and allows faster mobilization and recovery. Therefore, the PRDS technique could be the first reduction choice for patients with irreducible fractures.

## Data Availability

The data and materials are available from the corresponding author on reasonable request.

## Abbreviations

PRDS: Percutaneous reduction with double screwdrivers
LOR: Limited open reduction
BMI: Body mass index
ASA: American society of anesthesiologists
AP: Anteroposterior
DVT: Deep vein thrombosis
